# Estimated Deaths Attributable to Excessive Alcohol Use Among US Adults Aged 20 to 64 Years, 2015 to 2019

**DOI:** 10.1001/jamanetworkopen.2022.39485

**Published:** 2022-11-01

**Authors:** Marissa B. Esser, Gregory Leung, Adam Sherk, Michele K. Bohm, Yong Liu, Hua Lu, Timothy S. Naimi

**Affiliations:** 1 Division of Population Health, National Center for Chronic Disease Prevention and Health Promotion, Centers for Disease Control and Prevention, Atlanta, Georgia; 2 Canadian Institute for Substance Use Research, University of Victoria, Victoria, Canada

## Abstract

**Importance:**

Alcohol consumption is a leading preventable cause of death in the US, and death rates from fully alcohol-attributable causes (eg, alcoholic liver disease) have increased in the past decade, including among adults aged 20 to 64 years. However, a comprehensive assessment of alcohol-attributable deaths among this population, including from partially alcohol-attributable causes, is lacking.

**Objective:**

To estimate the mean annual number of deaths from excessive alcohol use relative to total deaths among adults aged 20 to 64 years overall; by sex, age group, and state; and as a proportion of total deaths.

**Design, Setting, and Participants:**

This population-based cross-sectional study of mean annual alcohol-attributable deaths among US residents between January 1, 2015, and December 31, 2019, used population-attributable fractions. Data were analyzed from January 6, 2021, to May 2, 2022.

**Exposures:**

Mean daily alcohol consumption among the 2 089 287 respondents to the 2015-2019 Behavioral Risk Factor Surveillance System was adjusted using national per capita alcohol sales to correct for underreporting. Adjusted mean daily alcohol consumption prevalence estimates were applied to relative risks to generate alcohol-attributable fractions for chronic partially alcohol-attributable conditions. Alcohol-attributable fractions based on blood alcohol concentrations were used to assess acute partially alcohol-attributable deaths.

**Main Outcomes and Measures:**

Alcohol-attributable deaths for 58 causes of death, as defined in the Centers for Disease Control and Prevention’s Alcohol-Related Disease Impact application. Mortality data were from the National Vital Statistics System.

**Results:**

During the 2015-2019 study period, of 694 660 mean deaths per year among adults aged 20 to 64 years (men: 432 575 [66.3%]; women: 262 085 [37.7%]), an estimated 12.9% (89 697 per year) were attributable to excessive alcohol consumption. This percentage was higher among men (15.0%) than women (9.4%). By state, alcohol-attributable deaths ranged from 9.3% of total deaths in Mississippi to 21.7% in New Mexico. Among adults aged 20 to 49 years, alcohol-attributable deaths (44 981 mean annual deaths) accounted for an estimated 20.3% of total deaths.

**Conclusions And Relevance:**

The findings of this cross-sectional study suggest that an estimated 1 in 8 total deaths among US adults aged 20 to 64 years were attributable to excessive alcohol use, including 1 in 5 deaths among adults aged 20 to 49 years. The number of premature deaths could be reduced with increased implementation of evidenced-based, population-level alcohol policies, such as increasing alcohol taxes or regulating alcohol outlet density.

## Key Points

**Question:**

What is the estimated proportion of deaths among US adults aged 20 to 64 years attributable to excessive alcohol consumption, and are there differences by sex, age, and US state?

**Findings:**

The estimates in this cross-sectional study of 694 660 mean deaths per year between 2015 and 2019 suggest that excessive alcohol consumption accounted for 12.9% of total deaths among adults aged 20 to 64 years and 20.3% of deaths among adults aged 20 to 49 years. Among adults aged 20 to 64 years, the proportion of alcohol-attributable deaths to total deaths varied by state.

**Meaning:**

These findings suggest that an estimated 1 in 8 deaths among adults aged 20 to 64 years were attributable to excessive alcohol use and that greater implementation of evidence-based alcohol policies could reduce this proportion.

## Teaser

This cross-sectional study estimates the mean annual number of deaths attributable to excessive alcohol use among US adults aged 20 to 64 years overall; by sex, age group, and US state; and as a proportion of total deaths.

## Introduction

Excessive alcohol use is associated with several leading causes of death among adults aged 20 to 64 years in the US, including heart disease, cancer, unintentional injury, and liver disease.^1^ Excessive alcohol use is a leading preventable cause of premature death,^2^ and rates of deaths due to fully alcohol-attributable causes (eg, alcoholic liver disease) have increased in the past decade, including among adults aged 20 to 64 years.^3^ However, a US-based assessment of alcohol-attributable deaths among this population that also accounts for partially alcohol-attributable causes (eg, cancers) is lacking. Using the conditions in the Centers for Disease Control and Prevention’s Alcohol-Related Disease Impact (ARDI) application,^4^ this study estimated the mean annual number of deaths due to excessive alcohol use among adults aged 20 to 64 years overall; by sex, age group, and US state; and as a proportion of total deaths.

## Methods

Mean annual national and state mortality data from January 1, 2015, to December 31, 2019, were obtained from the National Vital Statistics System, WONDER,^5^ and the ARDI application for the 58 alcohol-related causes. In addition to deaths due to fully alcohol-attributable causes, deaths due to partially alcohol-attributable conditions were calculated in the ARDI application using cause-specific, alcohol-attributable fractions (AAFs) for select acute (eg, injuries) and chronic (eg, cancers) conditions (eTable 1 in the Supplement). Deaths due to acute conditions were calculated using direct AAFs based on high blood alcohol concentrations (eg, ≥0.10 g/dL). Deaths due to 23 chronic conditions were calculated using indirect AAFs, which include the prevalence of mean daily alcohol consumption levels and cause-specific relative risks that corresponded to those consumption levels.^6^ To account for substantial underreporting of alcohol consumption in nationwide surveys, the same methodology as in the ARDI application was used to adjust alcohol consumption from 2 089 287 respondents to the 2015-2019 Behavioral Risk Factor Surveillance System.^6^ Consumption was adjusted to 73% of national per capita alcohol sales (from tax and shipment data in the Alcohol Epidemiologic Data System) to align with alcohol use reported in US epidemiologic cohort studies.^7^ This study estimated deaths due to excessive alcohol consumption; therefore, for chronic conditions, the adjusted prevalence of medium (>1 to ≤2 alcoholic drinks for women or >2 to ≤4 drinks for men) and high (>2 alcoholic drinks for women or >4 drinks for men) mean daily alcohol consumption (eTable 2 in the Supplement) were applied to relative risks to generate cause-specific AAFs.^4^ Alcohol-attributable fractions and relative risks are generally not available by race and ethnicity, and alcohol attribution to deaths might differ across these groups; therefore, deaths in this study were not estimated by race and ethnicity. Because data were deidentified and secondary analyses were performed, institutional review board oversight and informed consent were not required as determined by the Centers for Disease Control and Prevention under 45 CFR 46. This study followed the Strengthening the Reporting of Observational Studies in Epidemiology (STROBE) reporting guideline.

Analyses of alcohol consumption prevalence were conducted using SAS, version 9.4 (SAS Institute Inc). We used the ARDI application to assess the mean annual number of deaths due to excessive drinking and the leading causes of death.^4^ Alcohol-attributable deaths were calculated as a percentage of total deaths overall and by sex, age, and US state. Alcohol-attributable death rates per 100 000 population were assessed using US Census population counts from WONDER.^5^

## Results

Our findings suggest that an estimated annual mean of 140 557 deaths (men: 97 182 [69.1%]; women: 43 375 [30.9%]) could be attributed to excessive alcohol consumption in the US during the 2015-2019 study period, accounting for 5.0% of total deaths (Table 1). Among all adults aged 20 to 64 years, 694 660 annual mean total deaths were noted (men: 432 575 [66.3%]; women: 262 085 [37.7%]), and an estimated 89 697 of these (12.9%) were alcohol-attributable (64 998 [15.0%] among men and 24 699 [9.4%] among women). Our analysis showed that although the number and rate of alcohol-attributable deaths per 100 000 increased by age group, alcohol-attributable deaths accounted for a larger proportion of total deaths among younger groups: 19 782 of 77 973 total deaths (25.4%) among adults aged 20 to 34 years and 25 199 of 143 663 (17.5%) among those aged 35 to 49 years. The 3 leading causes of alcohol-attributable deaths by age group were the same for men and women (eg, adults aged 20-34 years: other poisonings, motor vehicle traffic crashes, and homicide; adults aged 35-49 years: other poisonings, alcoholic liver disease, and motor vehicle traffic crashes).

**Table 1.** Mean Annual Total and Estimated Alcohol-Attributable Deaths in the US, 2015 to 2019^a^

Sex by age group, y	Total No. of all-cause deaths	Deaths due to excessive alcohol use^a^		
		No. of deaths (%)^b^	Rate per 100 000 population	3 Leading causes (No. of deaths)^c^
Men and women				
All	2 792 885	140 557 (5.0)	43.2	Alcoholic liver disease (n = 22 472); other poisonings (n = 17 671); and motor vehicle traffic crashes (n = 12 650)
20-64	694 660	89 697 (12.9)	46.7	Alcoholic liver disease (n = 17 129); other poisonings (n = 16 609); and motor vehicle traffic crashes (n = 10 627)
20-34	77 973	19 782 (25.4)	29.4	Other poisonings (n = 5537); motor vehicle traffic crashes (n = 4707); and homicide (n = 3911)
35-49	143 663	25 199 (17.5)	40.8	Other poisonings (n = 6085); alcoholic liver disease (n = 4605); and motor vehicle traffic crashes (n = 3100)
50-64	473 024	44 716 (9.5)	70.8	Alcoholic liver disease (n = 11 709); other poisonings (n = 4987); and unspecified liver cirrhosis (n = 3878)
Men				
All	1 429 008	97 182 (6.8)	60.7	Alcoholic liver disease (n = 15 614); other poisonings (n = 12 014); and motor vehicle traffic crashes (n = 9590)
20-64	432 575	64 998 (15.0)	68.0	Alcoholic liver disease (n = 11 601); other poisonings (n = 11 337); and motor vehicle traffic crashes (n = 8227)
20-34	55 306	15 147 (27.4)	44.2	Other poisonings (n = 3990); motor vehicle traffic crashes (n = 3607); and homicide (n = 3323)
35-49	89 723	17 846 (19.9)	58.1	Other poisonings (n = 4131); alcoholic liver disease (n = 2945); and motor vehicle traffic crashes (n = 2395)
50-64	287 546	32 005 (11.1)	104.3	Alcoholic liver disease (n = 8155); other poisonings (n = 3216); and unspecified liver cirrhosis (n = 2395)
Women				
All ages	1 363 877	43 375 (3.2)	26.3	Alcoholic liver disease (n = 6857); hypertension (n = 6808); and other poisonings (n = 5656)
20-64	262 085	24 699 (9.4)	25.6	Alcoholic liver disease (n = 5527); other poisonings (n = 5273); and motor vehicle traffic crashes (n = 2399)
20-34	22 667	4635 (20.4)	14.0	Other poisonings (n = 1547); motor vehicle traffic crashes (n = 1100); and homicide (n = 588)
35-49	53 940	7353 (13.6)	23.7	Other poisonings (n = 1955); alcoholic liver disease (n = 1660); and motor vehicle traffic crashes (n = 704)
50-64	185 478	12 711 (6.9)	39.1	Alcoholic liver disease (n = 3553); other poisonings (n = 1771); and unspecified liver cirrhosis (n = 1482)

^a^ Consistent with the Centers for Disease Control and Prevention’s Alcohol-Related Disease Impact (ARDI) application,^4^ data pertain to excessive alcohol use, which includes deaths due to (1) conditions that are 100% alcohol-attributable, (2) acute conditions that involved binge drinking, and (3) chronic conditions that involved medium (>1 to ≤2 alcoholic drinks for women or >2 to ≤4 drinks for men) or high (>2 alcoholic drinks for women or >4 drinks for men) levels of mean daily alcohol consumption. Numbers may not sum to totals due to rounding.

^b^ For underlying chronic causes of death that were estimated in the ARDI application using indirect alcohol-attributable fractions that account for the risk of dying at 3 levels of mean daily alcohol consumption, self-reported mean daily alcohol consumption was adjusted to account for 73% of per capita alcohol sales.^7^

^c^ Other poisonings (eg, drug overdoses) indicates deaths involving another substance in addition to a high blood alcohol concentration (≥0.10 g/dL).

By state, alcohol-attributable deaths among adults aged 20 to 64 years ranged from 9.3% of total deaths in Mississippi to 21.7% in New Mexico (Table 2). State-level variations were found by age group (eg, the proportion of alcohol-attributable deaths to total deaths among adults aged 20-34 years ranged from 22.4% in Utah to 33.3% in New Mexico). Among adults aged 20 to 49 years, our estimates suggest that excessive drinking was responsible for 44 981 mean annual deaths, or 20.3% of total deaths. This percentage was generally lower in states in the Southeast and higher in the West, upper Midwest, and New England (Figure).

**Table 2.** Estimated Alcohol-Attributable Deaths and Percentage of Total Deaths Among Adults Aged 20 to 64 Years by State and Age Group

Location	Age group, No. of deaths due to excessive alcohol use (% of total deaths)^a^			
	20-34 y	35-49 y	50-64 y	20-64 y
Entire US	19 782 (25.4)	25 199 (17.5)	44 716 (9.5)	89 697 (12.9)
State^b^				
Alabama	381 (24.2)	443 (13.8)	665 (6.5)	1489 (9.9)
Alaska	86 (29.6)	97 (26.3)	149 (14.5)	332 (19.7)
Arizona	512 (27.3)	707 (22.5)	1148 (12.2)	2367 (16.4)
Arkansas	195 (23.2)	266 (14.6)	426 (7.1)	887 (10.3)
California	1880 (25.8)	2578 (19.2)	5051 (11.6)	9509 (14.8)
Colorado	390 (28.5)	536 (24.2)	902 (14.0)	1828 (18.2)
Connecticut	195 (26.6)	262 (19.8)	466 (10.5)	923 (14.2)
Delaware	78 (27.7)	89 (19.1)	148 (9.8)	315 (13.9)
Florida	1328 (25.6)	1700 (18.0)	3407 (10.6)	6435 (13.7)
Georgia	620 (23.5)	721 (14.0)	1272 (7.7)	2613 (10.7)
Hawaii	56 (23.0)	82 (15.8)	162 (9.2)	300 (11.9)
Idaho	87 (25.1)	129 (20.2)	240 (11.5)	456 (14.8)
Illinois	796 (27.1)	923 (17.7)	1558 (8.7)	3277 (12.6)
Indiana	465 (24.7)	575 (16.7)	930 (8.3)	1970 (11.9)
Iowa	133 (24.0)	202 (17.9)	409 (9.4)	744 (12.3)
Kansas	158 (24.1)	197 (16.8)	374 (8.6)	729 (11.8)
Kentucky	317 (23.1)	476 (15.7)	733 (7.8)	1526 (11.1)
Louisiana	408 (25.8)	454 (16.0)	716 (7.8)	1578 (11.6)
Maine	78 (26.1)	121 (20.0)	226 (10.2)	425 (13.6)
Maryland	467 (27.1)	476 (17.2)	755 (8.6)	1698 (12.8)
Massachusetts	397 (25.2)	503 (19.4)	867 (10.5)	1767 (14.3)
Michigan	626 (24.4)	799 (17.0)	1442 (8.8)	2867 (12.1)
Minnesota	230 (24.1)	334 (19.2)	680 (10.8)	1244 (13.8)
Mississippi	217 (22.5)	247 (12.2)	423 (6.4)	887 (9.3)
Missouri	492 (26.9)	551 (17.6)	865 (8.1)	1908 (12.2)
Montana	83 (30.0)	113 (24.5)	194 (12.1)	390 (16.7)
Nebraska	88 (25.9)	120 (17.6)	220 (9.0)	428 (12.3)
Nevada	178 (25.5)	278 (19.2)	525 (11.5)	981 (14.6)
New Hampshire	91 (25.6)	119 (20.8)	218 (11.2)	428 (14.9)
New Jersey	480 (24.6)	577 (16.6)	910 (8.1)	1967 (11.8)
New Mexico	261 (33.3)	383 (29.1)	530 (16.1)	1174 (21.7)
New York	817 (22.6)	1060 (15.6)	1965 (8.2)	3842 (11.2)
North Carolina	673 (25.3)	819 (16.3)	1414 (8.6)	2906 (12.1)
North Dakota	56 (27.6)	64 (21.4)	110 (11.9)	230 (16.1)
Ohio	871 (24.9)	1099 (17.3)	1791 (8.6)	3761 (12.3)
Oklahoma	273 (24.6)	400 (17.2)	724 (9.4)	1397 (12.6)
Oregon	203 (26.2)	336 (21.6)	756 (13.3)	1295 (16.1)
Pennsylvania	942 (25.8)	992 (16.9)	1690 (8.4)	3624 (12.2)
Rhode Island	59 (27.4)	90 (21.7)	160 (11.1)	309 (14.9)
South Carolina	387 (25.9)	470 (16.4)	840 (9.0)	1697 (12.4)
South Dakota	66 (30.6)	91 (24.2)	142 (12.2)	299 (17.0)
Tennessee	470 (23.1)	651 (15.4)	1120 (8.3)	2241 (11.4)
Texas	1608 (25.8)	1946 (16.3)	3416 (9.2)	6970 (12.6)
Utah	163 (22.4)	216 (18.7)	278 (10.2)	657 (14.3)
Vermont	35 (24.7)	45 (19.3)	99 (11.0)	179 (14.0)
Virginia	454 (25.2)	533 (15.8)	981 (8.3)	1968 (11.6)
Washington	355 (25.2)	512 (19.5)	1062 (11.6)	1929 (14.6)
Washington, DC	61 (30.5)	63 (18.5)	133 (11.6)	257 (15.2)
West Virginia	152 (23.9)	232 (16.7)	345 (8.1)	729 (11.6)
Wisconsin	326 (26.4)	415 (19.0)	849 (10.9)	1590 (14.2)
Wyoming	45 (29.5)	78 (27.6)	121 (14.3)	244 (19.0)

^a^ Mean annual number of alcohol-attributable deaths between January 1, 2015, and December 31, 2019. Numbers may not sum to totals due to rounding. Consistent with the Centers for Disease Control and Prevention’s Alcohol-Related Disease Impact (ARDI) application,^4^ data pertain to excessive alcohol use, which includes deaths due to (1) conditions that are 100% alcohol-attributable, (2) acute conditions that involved binge drinking, and (3) chronic conditions that involved medium (>1 to ≤2 alcoholic drinks for women or >2 to ≤4 drinks for men) or high (>2 alcoholic drinks for women or >4 drinks for men) levels of mean daily alcohol consumption. For underlying chronic causes of death that were estimated in the ARDI application using indirect alcohol-attributable fractions that account for the risk of dying at 3 levels of mean daily alcohol consumption, self-reported mean daily alcohol consumption was adjusted to account for 73% of per capita alcohol sales.^7^

^b^ Ranges in percentage of total deaths by state were 22.4% to 33.3% for those aged 20 to 34 years; 12.2% to 29.1% for those aged 35 to 49 years; 6.4% to 16.1% for those aged 50 to 64 years; and 9.3% to 21.7% for those aged 20 to 64 years.

**Figure.**
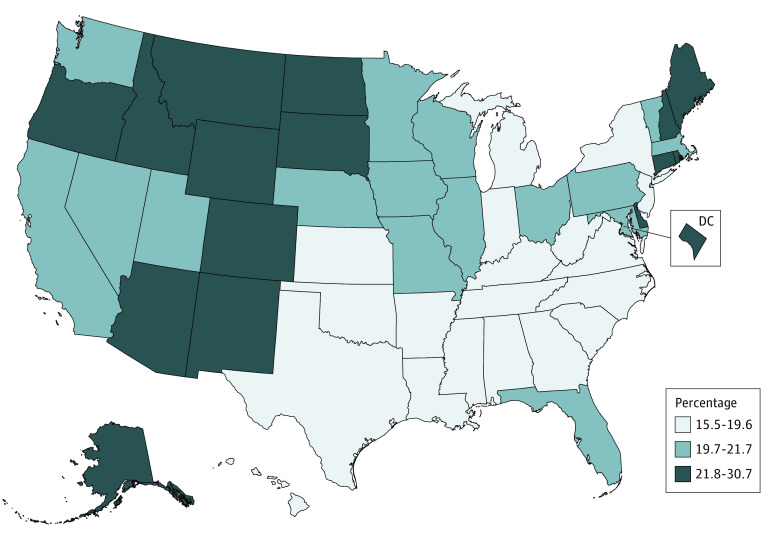
Estimated Percentage of Total Deaths Attributable to Excessive Alcohol Use Among US Adults Aged 20 to 49 Years, 2015 to 2019

## Discussion

We found that 89 697 of an estimated 140 557 deaths due to excessive alcohol use annually during the 2015-2019 study period, or nearly two-thirds of the deaths, were among adults aged 20 to 64 years. Our estimates suggest that alcohol-attributable deaths were responsible for 1 in 8 deaths among adults aged 20 to 64 years, including 1 in 5 deaths among adults aged 20 to 49 years.

Compared with 2019, death rates involving alcohol as an underlying or contributing cause of death increased during the first year of the COVID-19 pandemic in 2020, including among adults aged 20 to 64 years.^8^ Therefore, the proportion of deaths due to excessive drinking among total deaths might be higher than reported in this study. Nevertheless, these study findings are consistent with the epidemiology of excessive drinking. For example, the prevalence of binge drinking is generally higher among younger adults, and this population tends to consume more alcohol while binge drinking,^9^ which contributes to their leading causes of alcohol-attributable deaths.

The methods for estimating deaths due to excessive alcohol consumption in this study differ somewhat from those of other studies. From 2006 to 2010, an estimated 1 in 10 deaths among adults aged 20 to 64 years was attributable to excessive alcohol consumption.^10^ That finding was partially based on self-reported mean daily consumption prevalence estimates that were adjusted to account for binge drinking occasions but not per capita alcohol sales. Because survey-based adjustments alone can lead to underestimates of alcohol-attributable deaths that are calculated using indirect AAF methods, this study adjusted self-reported alcohol use data to account for 73% of per capita alcohol sales.^7^ Global studies estimating alcohol-attributable deaths also adjust using per capita alcohol sales, but they generally adjust to 80%.^11^ The ARDI methods used in this study provide estimates of deaths pertaining to excessive drinking rather than all levels of consumption. Also, the ARDI application uses direct AAFs for estimating the number of alcohol-attributable deaths due to acute causes. This method differs from those of global studies that estimate alcohol-attributable deaths across all levels of consumption and consistently base estimates on continuous risk functions.^11,12^

### Limitations

This study has some limitations. The alcohol-attributable death estimates in this study may be conservative because they are based on deaths due to alcohol-related conditions that were identified as the underlying cause of death only; contributing causes of death were not included. In addition, alcohol-attributable deaths due to partially alcohol-attributable conditions were not estimated for adults who formerly used alcohol, despite some dying of alcohol-related causes,^11^ because the prevalence of former alcohol consumption is not collected in the Behavioral Risk Factor Surveillance System. Direct AAFs were used to estimate alcohol-attributable deaths due to acute causes (eg, injuries)^6^; however, the sources of some AAFs were based on older data that may less accurately represent current alcohol-attribution. Last, some conditions related to alcohol use (eg, HIV/AIDS) were not included because suitable AAFs for the US were not available.

## Conclusions

The findings of this cross-sectional study suggest that an estimated 1 in 8 deaths among adults aged 20 to 64 years was attributable to excessive alcohol consumption, including 1 in 5 deaths among adults aged 20 to 49 years. These premature deaths could be reduced through increased implementation of evidence-based alcohol policies (eg, increasing alcohol taxes, regulating alcohol outlet density),^13^ and alcohol screening and brief intervention.^14^

## Notes

Supplement. **eTable 1.** *ICD-10* Codes and Alcohol-Attributable Fraction Information by Causes of Death in the Alcohol-Related Disease Impact Application **eTable 2.** Adjusted Prevalence of US Mean Daily Alcohol Consumption by Level of Consumption and Age Group
eReferences
