# Supplementary material for: Estimated Deaths Attributable to Excessive Alcohol Use Among US Adults Aged 20 to 64 Years, 2015 to 2019
Source: JAMA Netw Open. 2022 Nov 1;5(11):e2239485. doi: 10.1001/jamanetworkopen.2022.39485 (PMC9627409; doi:10.1001/jamanetworkopen.2022.39485)
Supplement: Supplement. — eTable 1. ICD-10 Codes and Alcohol-Attributable Fraction Information by Causes of Death in the Alcohol-Related Disease Impact Application eTable 2. Adjusted Prevalence of US Mean Daily Alcohol Consumption by Level of Consumption and Age Group eReferences [file jamanetwopen-e2239485-s001.pdf]

Supplementary Online Content

Esser MB, Leung G, Sherk A, et al. Estimated deaths attributable to excessive alcohol use among US adults aged 20 to 64 years, 2015 to 2019. *JAMA Netw Open*. 2022;5(11):e2239485. doi:10.1001/jamanetworkopen.2022.39485

eTable 1. *ICD-10* Codes and Alcohol-Attributable Fraction Information by Causes of Death in the Alcohol-Related Disease Impact Application
eTable 2. Adjusted Prevalence of US Mean Daily Alcohol Consumption by Level of Consumption and Age Group
eReferences

This supplementary material has been provided by the authors to give readers additional information about their work.

© 2022 Esser MB et al. *JAMA Network Open*.

eTable 1. *ICD-10* Codes and Alcohol-Attributable Fraction Information by Causes of Death in the Alcohol-Related Disease Impact Application

Cause of death	ICD-10 codes	AAF information
**Chronic conditions: Direct AAF**[a]		
Alcoholic psychosis	F10.3-F10.9	AAF = 1.0. No estimation required.
Alcohol abuse	F10.0, F10.1	AAF = 1.0. No estimation required.
Alcohol dependence syndrome	F10.2	AAF = 1.0. No estimation required.
Alcohol polyneuropathy	G62.1	AAF = 1.0. No estimation required.
Degeneration of nervous system due to alcohol	G31.2	AAF = 1.0. No estimation required.
Alcoholic myopathy	G72.1	AAF = 1.0. No estimation required.
Alcohol cardiomyopathy	I42.6	AAF = 1.0. No estimation required.
Alcoholic gastritis	K29.2	AAF = 1.0. No estimation required.
Alcoholic liver disease	K70.0-K70.4, K70.9	AAF = 1.0. No estimation required.
Alcohol-induced acute pancreatitis	K85.2	AAF = 1.0. No estimation required.
Alcohol-induced chronic pancreatitis	K86.0	AAF = 1.0. No estimation required.
Fetal alcohol syndrome	Q86.0	AAF = 1.0. No estimation required.
Fetus and newborn affected by maternal use of alcohol	P04.3	AAF = 1.0. No estimation required.
Esophageal varices	I85	AAF = 0.68. AAF calculated by summing all alcoholic liver cirrhosis deaths and 40% of the unspecified liver cirrhosis deaths[1] and chronic hepatitis not elsewhere classified, and then dividing that by the sum of total cirrhosis deaths (that is, all alcoholic liver cirrhosis deaths; all unspecified liver cirrhosis deaths; and all chronic hepatitis not elsewhere classified deaths).<br>Source AAF based on: Parrish et al. (1993)[1]
Gastroesophageal hemorrhage	K22.6	AAF = 0.47.<br>AAF source: English et al. (1995)[2]
Liver cirrhosis, unspecified	K74.0-K74.2, K74.6, K76.0, K76.7, K76.9	AAF = 0.40.<br>AAF source: Parrish et al. (1993)[1]
Portal hypertension	K76.6	AAF = 0.68. AAF calculated by summing all alcoholic liver cirrhosis deaths and 40% of the unspecified liver cirrhosis deaths[1] and chronic hepatitis not elsewhere classified, and then dividing that by the sum of total cirrhosis deaths (that is, all alcoholic liver cirrhosis deaths; all unspecified liver cirrhosis deaths; and all chronic hepatitis not elsewhere classified deaths).<br>Source AAF based on: Parrish et al. (1993)[1]
**Chronic conditions: Indirect AAF**[b]		
Atrial fibrillation	I48	Relative risk source: Samokhvalov et al. (2010a)[3]
Cancer, breast (females only)	C50	Relative risk source: Bagnardi et al. (2015)[4]
Cancer, colorectal	C18, C20	Relative risk source: Bagnardi et al. (2015)[4]
Cancer, esophageal	C15	Deaths calculated for the proportion of esophageal cancer deaths due to squamous

© 2022 Esser MB et al. *JAMA Network Open*.

		cell carcinoma only, based on the Surveillance, Epidemiology, and End Results data in 18 states (SEER18). Relative risk source: Bagnardi et al. (2015)[4]
Cancer, laryngeal	C32	Relative risk source: Bagnardi et al. (2015)[4]
Cancer, liver	C22	Relative risk source: Bagnardi et al. (2015)[4]
Cancer, oral cavity and pharynx	C01-C06, C09-C10, C12-C14	Relative risk source: Bagnardi et al. (2015)[4]
Cancer, pancreatic	C25	Deaths among people consuming high levels of alcohol only. Relative risk source: Bagnardi et al. (2015)[4]
Cancer, prostate (males only)	C61	AAF source: Bagnardi et al. (2015)[4]
Cancer, stomach	C16	Deaths among people consuming high levels of alcohol only. Relative risk source: Bagnardi et al. (2015)[4]
Chronic hepatitis	K73	Relative risk source: Corrao et al. (1999)[5]
Coronary heart disease	I20-I25	Relative risk source: Zhao et al. (2017)[6]
Gallbladder disease	K80, K81, K83	Relative risk source: English et al. (1995)[2]
Hypertension	I10-I13, I15	Relative risk source: Taylor et al. (2009)[7]
Infant death, low birth weight	P05.0, P07.0, P07.1	Alcohol consumption prevalence estimates calculated among females aged 18 to 44 years only. Relative risk source: Patra et al. (2011)[8]
Infant death, preterm birth	P07.2, P07.3	Alcohol consumption prevalence estimates calculated among females aged 18 to 44 years only. Relative risk source: Patra et al. (2011)[8]
Infant death, small for gestational age	P05.1	Alcohol consumption prevalence estimates calculated among females aged 18 to 44 years only. Relative risk source: Patra et al. (2011)[8]
Pancreatitis, acute	K85.0, K85.1, K85.3, K85.8, K85.9	Data were not available to determine the relative risk at the various levels of alcohol consumption; the AAF for acute pancreatitis for excessive consumption was calculated using the single relative risk estimate for both the medium and high mean daily alcohol consumption levels. Relative risk source: Alsamarrai et al. (2014)[9]
Pancreatitis, chronic	K86.1	Relative risk source: Corrao et al. (2004)[10]
Pneumonia	J12-J16, J18	Deaths from pneumonia were included for people aged 20 to 64 years only due to the high number of deaths from pneumonia among people aged 65 years and older that are not alcohol-related and the lack of relative risks that differ by age. Relative risk source: Samokhvalov et al. (2010b)[11]
Stroke, ischemic	G45, I63, I65-I67, I69.3	Relative risk source: Patra et al. (2010)[12]
Stroke, hemorrhagic	I60-I62, I69.0-I69.2	Relative risk source: Patra et al. (2010)[12]
Unprovoked seizures, epilepsy, or seizure disorder	G40, G41, R56.8	Relative risk source: Samokhvalov et al. (2010c)[13]

© 2022 Esser MB et al. *JAMA Network Open*.

**Acute conditions: Direct AAF**[a]		
Alcohol poisoning	X45, Y15	AAF = 1.0. No estimation required.
Suicide by and exposure to alcohol	X65	AAF = 1.0. No estimation required.
Air-space transport	V95-V97	AAF = 0.18.<br>AAF source: Smith et al. (1999)[14]
Aspiration	W78-W79	AAF = 0.18.<br>AAF source: Smith et al. (1999)[14]
Child maltreatment	X85-X99, Y00-Y09, Y87.1	AAF = 0.16.<br>Deaths among children aged 0 to 14 years.<br>AAF source: English et al. (1995)[2]
Drowning injuries	W65-W70, W73, W74, Y21	AAF = 0.34.<br>AAF source: Smith et al. (1999)[14]
Fall injuries	W00-W19, Y30	AAF = 0.32.<br>Deaths from falls were included for people aged 15 to 69 years only due to the high number of deaths from falls among persons aged 70 years and older that are not alcohol-related and the lack of AAFs that differ by age.<br>AAF source: Smith et al. (1999)[14]
Fire injuries	X00-X06, X08, X09, Y26	AAF = 0.42.<br>AAF source: Smith et al. (1999)[14]
Firearm injuries	W32-W34, Y22-Y24	AAF = 0.18.<br>AAF source: CDC National Violent Death Reporting System, 2015[15]
Homicide	X85-X99, Y00-Y09, Y87.1	AAF = 0.47. The AAF is from a meta-analysis that systematically reviewed studies that measured the blood alcohol levels of people who caused the crime (i.e., perpetrators) and not just the blood alcohol level of victims.<br>AAF source: English et al. (1995)[2]
Hypothermia	X31	AAF = 0.41.<br>AAF source: Smith et al. (1999)[14]
Motor-vehicle nontraffic crashes	V02.0, V03.0, V04.0, V09.0, V12-V14(.0-.2), V19.0-V19.2, V20-V28(.0-.2), V29.0-V29.3, V30-V39(.0-.3),V40-V49(.0-.3), V50-V59(.0-.3), V60-V69(.0-.3), V70-V79(.0-.3), V81.0, V82.0, V83-V86(.4-.7, .9), V88.0-V88.8, V89.0	AAF = 0.18.<br>AAF source: Smith et al. (1999)[14]
Motor-vehicle traffic crashes	V02(.1, .9), V03(.1, .9), V04(.1, .9), V09.2, V12-V14(.3-.5, .9), V19.4-V19.6, V20-V28(.3-.5, .9), V29.4-V29.6, V29.8,	AAF varies by sex, age group, and state, and available in the CDC Alcohol-Related Disease Impact application.[16] Deaths among people of all ages. A blood alcohol concentration level of 0.08 g/dL or greater is

© 2022 Esser MB et al. *JAMA Network Open*.

	V29.9, V30-V38(.4-.7, .9), V39 (.4-.6, .8, .9), V40-V48(.4-.7, 9), V49 (.4-.6, .8, .9), V50-V58(.4-.7, .9), V59 (.4-.6, .8, .9), V60-V68(.4-.7, .9), V69 (.4-.6, .8, .9), V70-V78(.4-.7, .9), V79 (.4-.6, .8, .9), V80.3-V80.5, V81.1, V82.1, V83-V86(.0-.3), V87.0-V87.8, V89.2	used for defining alcohol attribution for this condition.<br>AAF source: Fatality Analysis Reporting System, 2015-2019[17]
Occupational and machine injuries	W24-W31, W45	AAF = 0.18.<br>AAF source: Smith et al. (1999)[14]
Other road vehicle crashes	V01, V05-V06, V09.1, V09.3, V09.9, V10-V11, V15-V18, V19.3, V19.8-V19.9, V80.0-V80.2, V80.6-V80.9, V81.2-V81.9, V82.2-V82.9, V87.9, V88.9, V89.1, V89.3, V89.9	AAF = 0.18.<br>AAF source: Smith et al. (1999)[14]
Poisoning (not alcohol)	X40-X44, X46-X49, Y10-Y14, Y16-Y19	AAF = 0.29.<br>AAF source: Smith et al. (1999)[14]
Suicide	X60-X64, X66-X84, Y87.0	AAF = 0.24.<br>AAF source: CDC National Violent Death Reporting System, 2015[15]
Water transport	V90-V94	AAF = 0.18.<br>AAF source: Smith et al. (1999)[14]

AAF: Alcohol-attributable fraction, CDC: Centers for Disease Control and Prevention, ICD: International Classification of Diseases

[a] The number of alcohol-attributable deaths from conditions with a direct AAF was calculated by multiplying the total number of deaths from a particular cause by the AAF.

[b] Indirect AAFs vary by state as they were calculated using the state-specific prevalence of each mean daily alcohol consumption level and cause-specific relative risks that corresponded to those consumption levels. The relative risks by level of consumption and the state-specific AAFs are available in the CDC Alcohol-Related Disease Impact application.[16] The formula for calculating the AAF for excessive alcohol use in the Alcohol-Related Disease Impact application is as follows:

$$AAF_{EXC} = \frac{P_2(RS_2 - 1) + P_3(RS_3 - 1)}{1 + P_2(RS_2 - 1) + P_3(RS_3 - 1)}$$

Where:
P_2 is the prevalence of medium mean daily alcohol consumption
P_3 is the prevalence of high mean daily alcohol consumption
RR_1 is the relative risk corresponding to low mean daily alcohol consumption
RR_2 is the relative risk corresponding to medium mean daily alcohol consumption
RR_3 is the relative risk corresponding to high mean daily alcohol consumption
$RS_2 = \frac{RR_2}{RR_1}$ is the rescaled relative risk for medium mean daily alcohol consumption, using low mean daily alcohol consumption as the comparison group (following the methods of English et al.)[2]
$RS_3 = \frac{RR_3}{RR_1}$ is the rescaled relative risk for high mean daily alcohol consumption, using low mean daily alcohol consumption as the comparison group (following the methods of English et al.)[2]

© 2022 Esser MB et al. *JAMA Network Open*.

eTable 2. Adjusted Prevalence of US Mean Daily Alcohol Consumption by Level of Consumption and Age Group[a]

Characteristic	Level of mean daily alcohol consumption (Weighted %)			
	Nondrinking % (95% CI)	Low[b] % (95% CI)	Medium[c] % (95% CI)	High[d] % (95% CI)
**Total population, all ages**	**47.2 (47.1, 47.4)**	**35.4 (35.2, 35.5)**	**8.6 (8.6, 8.7)**	**8.8 (8.7, 8.8)**
20–64 years	43.0 (42.9, 43.2)	38.2 (38.0, 38.4)	9.5 (9.4, 9.6)	9.2 (9.1, 9.3)
20–34 years	38.6 (38.3, 38.9)	40.6 (40.3, 40.9)	11.0 (10.8, 11.2)	9.8 (9.7, 10.0)
35–49 years	42.6 (42.3, 42.9)	39.4 (39.1, 39.7)	9.3 (9.1, 9.4)	8.8 (8.6, 8.9)
50–64 years	47.9 (47.6, 48.2)	34.8 (34.5, 35.0)	8.3 (8.1, 8.4)	9.1 (8.9, 9.2)
**Men, all ages**	**41.2 (41.0, 41.4)**	**39.6 (39.4, 39.8)**	**9.6 (9.5, 9.7)**	**9.6 (9.5, 9.7)**
20–64 years	37.4 (37.2, 37.7)	42.4 (42.0, 42.4)	10.2 (10.1, 10.4)	10.1 (10.0, 10.3)
20–34 years	33.2 (32.7, 33.6)	44.7 (44.3, 45.2)	11.7 (11.4, 12.0)	10.5 (10.2, 10.7)
35–49 years	36.8 (36.4, 37.3)	43.4 (42.9, 43.8)	10.0 (9.7, 10.2)	9.8 (9.5, 10.1)
50–64 years	42.5 (42.1, 42.8)	38.5 (38.1, 38.8)	8.9 (8.7, 9.1)	10.1 (9.9, 10.4)
**Women, all ages**	**52.9 (52.7, 53.1)**	**31.4 (31.2, 31.6)**	**7.7 (7.6, 7.8)**	**8.0 (7.9, 8.1)**
20–64 years	48.5 (48.3, 48.7)	34.3 (34.1, 34.5)	8.8 (8.7, 8.9)	8.3 (8.2, 8.5)
20–34 years	44.1 (43.7, 44.6)	36.3 (35.9, 36.8)	10.3 (10.0, 10.6)	9.2 (9.0, 9.5)
35–49 years	48.3 (47.8, 48.7)	35.5 (35.1, 35.9)	8.6 (8.3, 8.8)	7.7 (7.5, 7.9)
50–64 years	52.9 (52.6, 53.3)	31.4 (31.0, 31.7)	7.6 (7.5, 7.8)	8.1 (7.9, 8.2)

CI: Confidence interval

[a] Weighted prevalence estimates are calculated among each subgroup (e.g., men *or* women) because this is the basis for applying prevalence estimates to the alcohol-attributable fraction formula, which is used for calculating alcohol-attributable deaths. Self-reported mean daily alcohol consumption from the 2015–2019 Behavioral Risk Factor Surveillance System was adjusted to account for 73% of per capita alcohol sales.

[b] Consumed a daily mean of >0–1 drink of alcohol (women) or >0–2 drinks (men).

[c] Consumed a daily mean of >1–2 drinks of alcohol (women) or >2–4 drinks (men).

[d] Consumed a daily mean of >2 drinks of alcohol (women) or >4 drinks (men).

© 2022 Esser MB et al. *JAMA Network Open*.

eReferences

1. Parrish KM, Dufour MC, Stinson FS, Harford TC. Average daily alcohol consumption during adult life among decedents with and without cirrhosis: the 1986 National Mortality Followback Survey. *J Stud Alcohol*. 1993;54(4):450-456. doi:10.15288/jsa.1993.54.450

2. English D, Holman C, Milne E, et al. *The Quantification of Drug Caused Morbidity and Mortality in Australia*. 1995.

3. Samokhvalov AV, Irving HM, Rehm J. Alcohol consumption as a risk factor for atrial fibrillation: a systematic review and meta-analysis. *Eur J Cardiovasc Prev Rehabil*. 2010;17(6):706-712. doi:10.1097/HJR.0b013e32833a1947

4. Bagnardi V, Rota M, Botteri E, et al. Alcohol consumption and site-specific cancer risk: a comprehensive dose-response meta-analysis. *Br J Cancer*. 2015;112(3):580-593. doi:10.1038/bjc.2014.579

5. Corrao G, Bagnardi V, Zambon A, Arico S. Exploring the dose-response relationship between alcohol consumption and the risk of several alcohol-related conditions: a meta-analysis. *Addiction*. 1999;94(10):1551-1573. doi:10.1046/j.1360-0443.1999.9410155111.x

6. Zhao J, Stockwell T, Roemer A, Naimi T, Chikritzhs T. Alcohol consumption and mortality from coronary heart disease: an updated meta-analysis of cohort studies. *J Stud Alcohol Drugs*. 2017;78(3):375-386. doi:10.15288/jsad.2017.78.375

7. Taylor B, Irving HM, Baliunas D, et al. Alcohol and hypertension: gender differences in dose-response relationships determined through systematic review and meta-analysis. *Addiction*. 2009;104(12):1981-1990. doi:10.1111/j.1360-0443.2009.02694.x

8. Patra J, Bakker R, Irving H, Jaddoe VW, Malini S, Rehm J. Dose-response relationship between alcohol consumption before and during pregnancy and the risks of low birthweight,

© 2022 Esser MB et al. *JAMA Network Open*.

preterm birth and small for gestational age (SGA)-a systematic review and meta-analyses. *BJOG*. 2011;118(12):1411-1421. doi:10.1111/j.1471-0528.2011.03050.x

9. Alsamarrai A, Das SL, Windsor JA, Petrov MS. Factors that affect risk for pancreatic disease in the general population: a systematic review and meta-analysis of prospective cohort studies. *Clin Gastroenterol Hepatol*. 2014;12(10):1635-1644. doi:10.1016/j.cgh.2014.01.038

10. Corrao G, Bagnardi V, Zambon A, La Vecchia C. A meta-analysis of alcohol consumption and the risk of 15 diseases. *Prev Med*. 2004;38:613-619. doi:10.1016/j.ypmed.2003.11.027

11. Samokhvalov AV, Irving HM, Rehm J. Alcohol consumption as a risk factor for pneumonia: a systematic review and meta-analysis. *Epidemiol Infect*. 2010;138(12):1789-1795. doi:10.1017/S0950268810000774

12. Patra J, Taylor B, Irving H, et al. Alcohol consumption and the risk of morbidity and mortality for different stroke types--a systematic review and meta-analysis. *BMC Public Health*. 2010;10:258. doi:10.1186/1471-2458-10-258

13. Samokhvalov AV, Irving H, Mohapatra S, Rehm J. Alcohol consumption, unprovoked seizures, and epilepsy: a systematic review and meta-analysis. *Epilepsia*. Jul 2010;51(7):1177-1184. doi:10.1111/j.1528-1167.2009.02426.x

14. Smith GS, Branas CC, Miller TR. Fatal nontraffic injuries involving alcohol: A metaanalysis. *Ann Emerg Med*. Jun 1999;33(6):659-668. doi:10.1016/S0749-3797(18)31070-5

15. Centers for Disease Control and Prevention. National Violent Death Reporting System (NVDRS). Accessed August 25, 2022. https://www.cdc.gov/violenceprevention/datasources/nvdrs/index.html

© 2022 Esser MB et al. *JAMA Network Open*.

16. Centers for Disease Control and Prevention. Alcohol-Related Disease Impact (ARDI) application. Accessed August 25, 2022. https://www.cdc.gov/ardi

17. National Highway Traffic Safety Administration. Fatality Analysis Reporting System (FARS). Accessed August 25, 2022. https://www.nhtsa.gov/research-data/fatality-analysis-reporting-system-fars

© 2022 Esser MB et al. *JAMA Network Open*.
